# IS*711*-based real-time PCR assay as a tool for detection of *Brucella *spp. in wild boars and comparison with bacterial isolation and serology

**DOI:** 10.1186/1746-6148-5-22

**Published:** 2009-07-14

**Authors:** Vladimira Hinić, Isabelle Brodard, Andreas Thomann, Milena Holub, Raymond Miserez, Carlos Abril

**Affiliations:** 1 National Centre for Zoonoses, Bacterial Animal Diseases and Antimicrobial Resistance (ZOBA), Institute of Veterinary Bacteriology, University of Bern, Vetsuisse Faculty, Länggass-Strasse 122, PO Box, CH-3001 Bern, Switzerland; 2 Amt für Lebensmittelsicherheit und Tiergesundheit, Planaterrastrasse 11, 7001 Chur, Switzerland

## Abstract

**Background:**

Control of brucellosis in livestock, wildlife and humans depends on the reliability of the methods used for detection and identification of bacteria. In the present study, we describe the evaluation of the recently established real-time PCR assay based on the *Brucella*-specific insertion sequence IS*711 *with blood samples from 199 wild boars (first group of animals) and tissue samples from 53 wild boars (second group of animals) collected in Switzerland. Results from IS*711 *real-time PCR were compared to those obtained by bacterial isolation, Rose Bengal Test (RBT), competitive ELISA (c-ELISA) and indirect ELISA (i-ELISA).

**Results:**

In the first group of animals, IS*711 *real-time PCR detected infection in 11.1% (16/144) of wild boars that were serologically negative. Serological tests showed different sensitivities [RBT 15.6%, c-ELISA 7.5% and i-ELISA 5.5%] and only 2% of blood samples were positive with all three tests, which makes interpretation of the serological results very difficult. Regarding the second group of animals, the IS*711 *real-time PCR detected infection in 26% of animals, while *Brucella *spp. could be isolated from tissues of only 9.4% of the animals.

**Conclusion:**

The results presented here indicate that IS*711 *real-time PCR assay is a specific and sensitive tool for detection of *Brucella *spp. infections in wild boars. For this reason, we propose the employment of IS*711 *real-time PCR as a complementary tool in brucellosis screening programs and for confirmation of diagnosis in doubtful cases.

## Background

Brucellosis is a widespread zoonosis of great economic importance caused by facultative intracellular Gram-negative bacteria belonging to the genus *Brucella*. Although brucellosis in domestic animals has been eradicated in great number of European countries, the risk of reintroduction of the disease still exists through spill-over from wildlife that are considered to be natural reservoirs [1]. A study on the surveillance of different swine pathogens demonstrated the presence of *Brucella suis *biovar 2 in a population of wild boars in Switzerland [2,3].

Reliable and sensitive diagnostic tools play a crucial role in the control of brucellosis in livestock, wildlife and humans. Although blood and tissue cultures remain the 'gold standard' for diagnosis, they show low sensitivity, are time consuming, and represent a risk for laboratory personnel [4,5].

Serology is a standard method for the epidemiological surveillance of brucellosis [2,3,6-9]. However, cross-reactions between *Brucella *species and other Gram-negative bacteria, such as *Yersinia enterocolitica *O:9, *Francisella tularensis*, *Escherichia coli *O:157, *Salmonella urbana *group N, *Vibrio cholerae *and *Stenotrophomonas maltophilia*, are a major problem of the serological assays [10-13]. The source of antigenic cross-reactions is the O-chain of the smooth lipopolysaccharide (S-LPS) present on the surface of the bacterial cell, which shows great similarity in smooth *Brucella *spp. and the abovementioned bacteria [14]. False-positive serological results due only to *Y. enterocolitica *O:9 affect up to 15% of the cattle herds in regions free from brucellosis, generating considerable additional costs for surveillance programs [13]. False-negative results have also been observed in serological diagnosis of brucellosis [11,15-17]. They occur mostly due to the fact that the antibody response is dependent upon the stage of infection during sample collection [18]. For example, Leal-Klevezas and colleagues stated that detectable amounts of antibodies are not recorded in the first 12–16 days after artificial inoculation of goats with *Brucella abortus *[19]. On the other hand, when the disease becomes chronic, the antibody titre could fall to undetectable levels [17,20], which is especially the case with intracellular organisms like *Brucella *spp. [21]. Latent infection without seroconversion further complicates the problem, particularly in pre-pubertal animals [22].

Molecular diagnostic techniques represent an important breakthrough in the diagnostic practice. A number of genus- or species-specific conventional PCR assays using primers derived from different gene sequences from the *Brucella *genome, such as 16S rRNA [23], the 16S-23S intergenic spacer region [24], *omp2 *[25] and *bcsp31 *[26], have been established. These assays were adapted for application to *Brucella *detection in different clinical specimens. In the majority of studies, conventional PCR proved to be a good means to detect *Brucella *DNA from clinical specimens [27-35], while Romero and colleagues found that PCR had lower sensitivity compared to the conventional detection methods [36].

The introduction of real-time PCR offers improved sensitivity, specificity and speed of performance compared with conventional PCR. Several real-time PCR assays using different detection chemistries have already been established for *Brucella *identification [37-39]. Moreover, some of them were evaluated with various clinical samples of human and animal origins [40-45]. Most of the authors confirmed that real-time PCR was a very sensitive method of detection from clinical samples [41,43,44]; nevertheless, O'Leary and colleagues found that there was no advantage in using real-time PCR on blood, milk and lymph node samples of naturally infected cows over standard serological and bacteriological methods [45].

The goal of our present study was to evaluate the performance of a recently described real-time PCR assay [46] for *Brucella *spp. detection with wild boar blood and tissue samples collected under the wild boar surveillance program in Switzerland. This assay is based on the *Brucella *spp. specific multiple IS*711 *insertion sequence and therefore shows great sensitivity [46]. The same samples were additionally tested by bacterial isolation and three serological tests (i-ELISA, c-ELISA and RBT), and the results obtained were compared to those of the real-time PCR.

## Results

### Bacteriological isolation

There were no differences in the results obtained with bacteriological isolation before and after freezing of the tissue samples. *Brucella *was isolated from tissue samples of 5 (9.4%) out of 53 animals (Table 1). According to the bacterial isolation method, the highest prevalence was found in tissues of reproductive organs (three isolates in uterus, one in accessory sexual glands, one in preputium). *Brucella *was also isolated from one spleen and one lung sample. All *Brucella *strains were identified as *B. suis *biovar 2. Table 2 shows the detection of *Brucella *spp. in individual tissue samples by bacterial isolation and IS*711 *real-time PCR. In three animals, bacterial colonies with morphological characteristics similar to *Brucella *spp. were isolated. Their lysates tested negative with the IS*711 *real-time PCR assay and were subsequently submitted for 16S rRNA gene sequence analysis. The sequence showed 99% identity with Bisgaard Taxon 10 (*Pasteurellaceae*).

**Table 1:** Detection of *Brucella *spp. in tissue samples by bacterial isolation and IS*711 *real-time PCR from wild boars.

		Positive samples	
		
Method	Number of animals	No.	%
Bacterial isolation	53	5	9.4
IS*711 *real-time PCR	53	14	26

**Table 2:** Detection of *Brucella *spp. in tissue samples by bacterial isolation and IS*711 *real-time PCR.

Tissue samples										
	Spleen		Acc. sex glands		Testicles		Uterus		Other	
					
Positive Animal	Bact. Isolation	IS711 RT-PCR	Bact. isolation	IS711 RT-PCR	Bact. isolation	IS711 RT-PCR	Bact. isolation	IS711 RT-PCR	Bact. isolation	IS711 RT-PCR

1	+	+					+	+		
2	-	+	+	+	-	+			+^a^	+^a^
3	-	+					+	+		
4	-	+	-	+	-	+				
5	-	+					-	+	+^b^	+^b^
6	-	-					-	+		
7	-	-					-	+		
8	-	+			-	+				
9	-	+							-^c^	+^c^
10	-	+	-	+	-	+				
11	-	-	-	+	-	-				
12	-	+	-	+	-	+			-^d^	+^d^
13	-	+					+	+		
14	-	+	-	+	-	+				

^a ^Preputium

^b ^Lungs

^c ^Placenta

^d ^Urine

No *Brucella *bacteria could be isolated from blood samples.

### Serological testing

**Figure 1:**
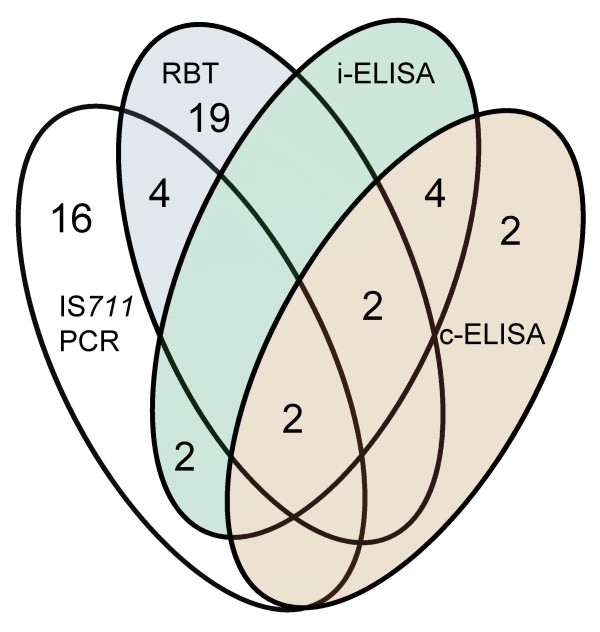
**Venn diagram showing a summary of serological (RBT, c-ELISA and i-ELISA) and IS*711 *real-time PCR results for blood samples from wild boars**. Footnote: In order to simplify the comparison, the six equivocal samples in i-ELISA, four blood samples for which the result was not obtained with RBT, and nine samples inhibited in the real-time PCR were not included in the comparison (total number of blood samples included into the comparison analysis n = 180).

Sera from 199 animals were tested with 3 different serological methods. RBT showed the highest sensitivity, detecting 31 (15.6%) positive samples while results could not be obtained for 4 (2%) samples due to strong haemolysis. With the c-ELISA, 15 (7.5%) samples tested positive. i-ELISA detected 11 (5.5%) positive samples, while 6 (3%) samples showed equivocal results. Table 3 summarises the results of the different methods used for testing the blood samples. Only 4 (2%) samples were found positive with all 3 serological tests. Figure 1 and Table 4 show the comparative analysis of positive samples detected with both serology and IS*711 *real-time PCR.

**Table 3:** Results of different methods for *Brucella *spp. diagnosis from blood samples in wild boars.

		Positive samples detected		Equivocal samples		No result^a^	
				
Method	Total Samples	No.	%	No.	%	No.	%
i-ELISA	199	11	5.5	6	3		
c-ELISA	199	15	7.5				
RBT	199	31	15.6			4	2
IS*711 *real-time PCR	199	27	13.6			9	4.5

^a ^The results could not be obtained for four samples tested with RBT due to strong haemolysis, and for nine samples tested with IS*711 *real-time PCR because of inhibition caused by their poor quality

### Testing of tissue samples with IS*711 *real-time PCR

The signal for 18S rRNA gene (endogenous extraction control) was detected for all samples. By testing tissue samples originating from 53 wild boars, all 5 positive (9.4%) animals detected by bacterial isolation were also detected by IS*711 *real-time PCR. Furthermore, with the IS*711 *real-time PCR, we were able to additionally detect nine (17%) infected animals that were negative by the bacterial isolation method. Interestingly, in 10 out of 14 positive animals, IS*711 *real-time PCR was able to detect *Brucella-*specific DNA in every organ that was available for examination. Table 1 shows the number of positive animals detected by bacterial isolation and IS*711 *real-time PCR. Table 2 shows the detection of *Brucella *spp. in individual tissue samples by bacterial isolation and IS*711 *real-time PCR. The quantification of bacteria per host cell in various organs revealed that of the 14 real-time PCR positive animals, 11 of them had the largest quantities of *Brucella *DNA in sexual organs (uterus, uterine fluid, testicle or accessory sexual gland), ranging up to 8081 IS*711 *copies pro 1000 cells. In two animals the spleen was principally affected (7 and 74.5 IS*711 *copies pro 1000 cells), whereas in only one animal the largest *Brucella *DNA quantity was found in urine (2.5 IS*711 *copies pro 1000 cells).

### Testing of blood samples with IS*711 *real-time PCR

As already mentioned, only leukocyte pellets were used for DNA extraction from blood samples. In 9 out of 199 (4.5%) samples, results were not obtained with the real-time PCR due to inhibition caused by the poor quality of the given blood samples. The signal for 18S rRNA gene (endogenous extraction control) was detected for all samples, except the nine aforementioned samples that were inhibited. Out of 199 blood samples, 27 (13.6%) tested positive with the IS*711 *real-time PCR. Surprisingly, IS*711 *real-time PCR detected infection in 11.1% (16/144) of wild boars that were serologically negative. Table 3 summarises the results of the different methods used for testing blood samples.

## Discussion

In this study, we report the performance of the recently described real-time PCR assay for the detection of *Brucella *spp. [46] in blood and tissue samples from naturally infected wild boars. Results obtained were compared with the results of bacterial isolation and three different serological tests for detection of brucellosis: RBT, i-ELISA and c-ELISA. This real-time PCR assay is very appealing as a diagnostic tool because it targets the IS*711 *insertion element, which is present in multiple copies in *Brucella *genomes and at the same time represents a stable genetic element with respect to number and positions in the genomes of various *Brucella *species [47,48]. The IS*711 *real-time PCR assay has been shown to be specific for *Brucella *spp. with a detection limit of 10 copies, indicating high assay sensitivity [46].

Regarding *Brucella *spp. detection in wild boar tissue samples, the IS*711 *assay was able to additionally detect *Brucella *DNA in tissues of 9 (17%) animals that were negative by bacterial isolation (Table 1 and 2). This low sensitivity of the culture method has already been reported by different authors [4,5]. The significantly higher detection sensitivity of real-time PCR can be explained by the fact that it detects DNA from bacteria that are damaged and nonviable and therefore impossible to isolate by conventional cultures. In 10 out of 14 real-time PCR positive animals, the IS*711 *real-time PCR assay was able to detect *Brucella *DNA from almost every organ that was submitted for examination (Table 2). The quantification of bacteria per host cell in various organs revealed that in 11 of the real-time PCR positive animals, the largest quantities of *Brucella *DNA were found in sexual organs, in two animals the spleen was principally affected, whereas in only one animal the largest *Brucella *DNA quantity was found in urine. These differences probably correlated with the stage of infection in individual animals. Since the samples at our disposition originated from animals shot by hunters, it was impossible to compare the variation in sensitivity of the different methods during the course of infection, according to clinical status or age.

No *Brucella *bacteria could be isolated from blood samples. This is in concordance with the reports of other authors stating that recovery of the bacteria by blood and milk culture is insensitive [19].

In contrast, IS*711 *real-time PCR was able to detect Brucella DNA in 27 (13.6%) out of 199 blood samples (Table 3). It is relevant to mention that the PCR performance with the *Brucella *DNA extracted from blood samples is very often compromised by the presence of PCR inhibitors and further complicated because *Brucella *is an intracellular pathogen [27]. However, the protocols presented here make detection of *Brucella *DNA from blood samples more feasible because only the leukocyte pellet was used for DNA extraction. Although it was reported that high concentrations of leukocyte DNA could inhibit the PCR assay [49], the IPC (internal positive control) signal was detected for all samples used in this assay except for nine samples, which were inhibited due to poor blood sample quality.

It is interesting to remark the discrepancies with the serological results. Comparing serology with the results of the IS*711 *assay, only 2 (1.1%) out of 180 samples included in the comparative analysis were positive with all serological tests and IS*711 *real-time PCR (Figure 1 and Table 4). Interestingly, IS*711 *real-time PCR detected infection in 11.1% (16/144) of the seronegative wild boars (Figure 1 and Table 4), which prompts us to conclude that these were probably acute or chronically infected animals with antibody levels beyond the detectable limit. Latent infection without seroconversion further complicates the problem, particularly in pre-pubertal animals [22]. While PCR directly detects the DNA of the pathogen, the serology is dependent upon the variable titres of antibodies in different phases of the disease [18]. On the other hand, 18% (28/156) of IS*711 *real-time PCR negative samples were seropositive in our study, which could be either due to a lack of sensitivity of the real-time PCR technique or serological false positives. Keeping in mind the well documented problem of extensive serological cross-reactions with other bacteria [10,12,13,22], it is highly likely that a great number of these samples were indeed false positives. Further difficulties associated with serological testing in wildlife is the fact that most tests have been directly transposed, without validation, from their use in domestic animals to the wild species, even though they may not perform identically [22].

**Table 4:** *Brucella *spp. positive samples detected with IS*711 *real-time PCR and serological tests.

	Positive samples detected	
	
Method	No.	%
IS*711 *real-time PCR (only)	16	8.9
IS*711 *real-time PCR and one serological test	6	3.3
IS*711 *real-time PCR and two serological tests	-	-
IS*711 *real-time PCR and three serological tests	2	1.1

In order to simplify the comparison, the six equivocal samples in i-ELISA, four blood samples for which the result was not obtained with RBT, and nine samples inhibited in the real-time PCR were not included in the comparison (total number of blood samples included into the comparison analysis n = 180).

In summary, in this study, the IS*711 *real-time PCR was able to increase the number of positive animals which were negative by bacterial isolation and detect additional positive animals that were seronegative. Moreover, not all the seropositive animals were detected positive by the IS*711 *real-time PCR. Therefore, it is important to use more than one type of diagnostic techniques for the detection of brucellosis in animals, which is an issue that has already been addressed by different authors [50,51].

## Conclusion

The IS*711 *assay described here is a sensitive and specific method for detection of *Brucella *spp. in blood and tissue specimens of wild boars. Since the data on prevalence of brucellosis in wild boar populations is still being estimated by serology [2,3,6-9], we are of the opinion that this assay should be included in brucellosis screening programs in order to complement the drawbacks of the conventional detection methods. Additionally, this assay should also be a method of choice for diagnosis of brucellosis in various wild and zoo animals, considering that current serological tests are evaluated only for domestic animals.

## Methods

### Study design and collection of clinical specimens

The clinical material to be tested in this study originated from a population of wild boars in Switzerland that, as demonstrated by Leuenberger and colleagues, represents a natural reservoir of *Brucella suis *biovar 2 [2]. The tissue and blood samples collected do not originate from the same animals and were collected under the national surveillance program for infectious diseases for wild boars, organized by the Swiss Federal Veterinary Office. The first group of samples consisted of various organs originating from 53 animals: spleen, testicles (tissue samples from both testicles of each boar were examined separately), accessory sexual glands, uteri in different stages of gravidity as well as non-gravid uteri samples, lung, and in individual cases penis with prepuce, placenta, kidney, and bladder containing urine. The organs were collected during the hunting seasons of 2001/2002 and 2002/2003 and stored at -20°C. Bacterial isolation from the organs was immediately done after the samples arrived to our laboratory. However, and in order to compare bacterial isolation results with these of the real-time PCR, bacterial isolation experiments were repeated and done simultaneously with the real-time PCR three to five years later. The second group of clinical samples consisted of 199 blood samples (in some animals a sero-sanguineous fluid from the thoracic cavity was taken), each from one animal. The sera were separated and examined by serological tests while the remaining blood clots were frozen at -20°C for one to two years and used for bacterial cultivation and DNA extraction for IS*711 *real-time PCR analysis.

### Bacteriological isolation

Tissue samples were thawed, the surfaces were heat sterilised and an internal sub-sample was inoculated onto three different nutritive media: Tryptic Soy Agar (TSA) plates containing 5% sheep blood (Oxoid, Basingstoke, Hampshire, England), Brolac agar (BioMérieux, Genève, Switzerland), and *Brucella *agar. *Brucella *agar was made from *Brucella *medium base (Oxoid) containing 5% inactivated horse serum with Modified *Brucella *Selective Supplement (Oxoid, SR0209E), as described in the OIE Manual of Standards for Diagnostic Tests and Vaccines, 2008 [52]. Brolac agar was used in order to facilitate the identification of contaminants growing on the blood plate, which, as expected, were very abundant because the samples were partially autolytic. The cultures were incubated at 37°C under an atmosphere with 5% CO_2_. Growth and morphology of the colonies were monitored on the second and fifth days after inoculation. For cultivation of blood samples, the leukocyte pellet obtained from 300 μl of blood clots (semi-liquid consistency) was inoculated on the plates. Only blood samples that tested positive with the IS*711 *real-time PCR were used for bacterial cultivation. Suspicious colonies were identified as *Brucella *spp. based on morphological, cultural and biochemical characteristics, such as oxidase and urease tests. The final species and biovar differentiations were done at the French OIE Reference Laboratory (Agence Française de Sécurité Sanitaire des Aliments, Cedex).

### Serological testing

Sera were tested for antibodies against *Brucella *using competitive ELISA (SVANOVIR^® ^*Brucella*-Ab c-ELISA, Svanova Biotech AG Uppsala, Sweden), indirect ELISA (CHEKIT^® ^*Brucella suis*, Dr. Bommeli AG/Idexx, Switzerland) and Rose Bengal spot agglutination test (RBT) as recommended by the OIE Manual of Standards for Diagnostic Tests and Vaccines, 2008 [52]. The cut-off values for the c-ELISA and i-ELISA were determined according to the manufacturers' guidelines. Although routinely performed at our institute, complement fixation test (CFT) is not adequate as a confirmatory test for haemolytic sera of wild boars from the hunting bag and therefore could not be used for these samples.

### DNA extraction from clinical samples

DNA from 25 to 30 mg of tissue (or 10 mg of spleen) and 100 μl of liquid samples, such as amniotic fluid and urine, were extracted using the QIAamp^® ^DNA Mini Kit (QIAGEN, Basel, Switzerland) according to the manufacturer's tissue protocol. For the DNA extraction from blood samples, only the leukocyte pellet was used. Briefly, 300 μl of blood clots (semi-fluid consistency) was resuspended in 600 μl erythrocyte lysis buffer (1.55 M NH_4_Cl, 0.1 M KHCO_3_, 1 mM Titriplex III, 10× conc.) and 300 μl PBS buffer. The suspension was mixed, incubated for 10 minutes and centrifuged at 8000 rpm for 2 minutes. The supernatant was discarded and the treatment with erythrocyte lysis solution was repeated until the leukocyte pellets lost all reddish colouring. The DNA from the leukocyte pellet was extracted following the tissue protocol of the QIAamp^® ^DNA Mini Kit (QIAGEN). The TaqMan^® ^Ribosomal RNA Control Reagent (Applied Biosystems, Foster City, CA, USA) designed to detect the 18S ribosomal RNA (rRNA) gene highly conserved among a diverse group of eukaryotes was used as an endogenous control of DNA extraction.

### IS*711 *real-time PCR

The primers and TaqMan^® ^probe selected from the sequences of the IS*711 *element and used for amplification and detection were described previously by Hiniæ and colleagues [46]. Briefly, real-time amplifications were performed using 2.5 μl of DNA extract, 300 nM of each primer, 200 nM probe and TaqMan^® ^Universal PCR Master Mix, No AmpErase^® ^UNG (Applied Biosystems) in a 25-μl volume. An exogenous Internal Positive Control (IPC, Applied Biosystems) was added to each reaction, according to the manufacturer's protocol, in order to check for the presence of PCR inhibitors. Amplification and real-time fluorescence detection was performed on a TaqMan^® ^7500 Real-time PCR system (Applied Biosystems) according to the standard protocol. A positive result was indicated by fluorescence above a threshold of 0.06 with the auto settings used for the baseline.

### Quantification of bacteria

The TaqMan^® ^Ribosomal RNA Control Reagent (Applied Biosystems) designed to detect the 18S ribosomal RNA (rRNA) gene was used as an endogenous extraction control and for quantification of host cells. The quantification of bacteria per host cell in various organs was done using the standard curve method (User's Manual, ABI PRISM 7700 Sequence Detection System, Applied Biosystems).

## Authors' contributions

VH carried out the experimental work, performed the analysis of the results and drafted the manuscript. IB and AT participated in the identification of bacterial isolates. MH performed RBT and i-ELISA. RM participated in the design of the study and helped in the interpretation of the results. CA conceived the study, participated in its design and coordination, and helped to draft the manuscript. All authors read and approved the final manuscript.
